# Analysis of Selected Variants of *DRD2* and *ANKK1* Genes in Combat Athletes

**DOI:** 10.3390/genes12081239

**Published:** 2021-08-13

**Authors:** Monika Michałowska-Sawczyn, Krzysztof Chmielowiec, Jolanta Chmielowiec, Grzegorz Trybek, Jolanta Masiak, Marta Niewczas, Paweł Cieszczyk, Wojciech Bajorek, Paweł Król, Anna Grzywacz

**Affiliations:** 1 Faculty of Physical Culture, Gdansk University of Physical Education and Sport, K. Górskiego St. 1, 80-336 Gdansk, Poland; monikamichalowska@op.pl (M.M.-S.); cieszczyk@poczta.onet.pl (P.C.); 2 Department of Hygiene and Epidemiology, Collegium Medicum, University of Zielona Góra, 28 Zyty St., 65-046 Zielona Góra, Poland; chmiele@vp.pl (K.C.); chmiele1@o2.pl (J.C.); 3 Department of Oral Surgery, Pomeranian Medical University in Szczecin, 72 Powstańców Wlkp. St., 70-111 Szczecin, Poland; g.trybek@gmail.com; 4 Neurophysiological Independent Unit, Department of Psychiatry, Medical University of Lublin, 1 Aleje Racławickie St., 20-059 Lublin, Poland; jolantamasiak@wp.pl; 5 College of Medical Sciences, Institute of Physical Culture Studies, University of Rzeszow, St. Towarnickiego 3, 35-955 Rzeszów, Poland; mniewczas@ur.edu.pl (M.N.); wbajorek@ur.edu.pl (W.B.); pkrol@ur.edu.pl (P.K.); 6 Independent Laboratory of Health Promotion, Pomeranian Medical University, 11 Chlapowskiego St., 70-204 Szczecin, Poland

**Keywords:** physical effort, *DRD2*, *ANKK1*, MMA, polymorphisms

## Abstract

The level of physical activity is conditioned by many different factors, including, among others, the personality traits of a person. Important is the fact that personality traits are a moderately heritable factor and on the basis of the analysis of several genes, various lifetime outcomes can be predicted. One of the most important pathways influencing personality traits is connected to the dopaminergic system; hence, we decided to analyze the *DRD2* *PROM.* rs1799732, *DRD2* rs1076560, *DRD2 Tag1D* rs1800498, *DRD2* Ex8 rs6276, *DRD2Tag1B* rs1079597 and *ANKK1 Tag1A* rs180049. The research group included 258 male athletes (mean age = 26.02; SD = 8.30), whereas the control group was 284 healthy male volunteers matched for age (mean age = 22.89; SD = 4.78), both of Caucasian origin and without history of substance dependency or psychosis. Genomic DNA was extracted from venous blood using standard procedures. Genotyping was conducted with the real-time PCR method. Differences in the frequency of the *DRD2Tag1B* rs1079597 gene polymorphism were found between people practicing combat sports and the control group, and the *DRD2 PROM.* rs1799732, *DRD2* rs1076560, *DRD2 Tag1D* rs1800498, *DRD2 Ex8* rs6276, *DRD2Tag1B* rs1079597 and *ANKK1 Tag1A* rs1800497 genotypes and allele frequencies in the studied sample did not differ between the analyzed groups. Hence, we considered these polymorphic places as an interesting area for the further search for unambiguous associations between personality traits and attitude towards physical effort.

## 1. Introduction

Currently, physical activity seems to be treated as one of the most important elements in the prevention of diseases of civilization. However, there are several factors that can influence individual attitudes towards participation in physical activity and also the achievements of an individual. A factor of high importance for the creation of a sporting attitude and attainment of achievements is personality. There exist numerous publications emphasizing that personality traits are a moderately heritable factor, and based on this, various lifetime outcomes can be predicted, such as disease vulnerability or attitude towards physical activity [1,2,3].

Fischer Lee and coauthors indicate an interaction between genes and the environment [4] that makes the analysis more complex and demanding. Nevertheless, the influence of genes cannot be treated as a marginal factor; hence, we wanted to concentrate on the influence of one of the polymorphisms observed within the genes connected with dopamine secretion and distribution. To be more precise, the *DRD2* gene and rs1076560 polymorphism. Human DNA (deoxyribonucleic acid) is composed of two polynucleotide chains composed of simpler monomeric units called nucleotides [5,6]. Each nucleotide is composed of one of four nitrogen-containing nucleobases, which are cytosine (C), guanine (G), adenine (A) or thymine (T). Such structure allows us to observe some differences that are connected with the change of one nucleobase with another, called SNPs (single nucleotide polymorphisms). An SNP is a germline substitution of a single nucleotide at a specific position in the genome, that can result in a change in protein properties [7]. There is a difference between an SNP and an allele, that is, one of two or more versions of a gene. Every individual inherits two alleles for each gene, one from each parent. The consequence of both allelic and polymorphic differences can be functional differences in the protein that is synthesized [8]. Such differences can be of very high significance in sport achievements. Because genes are responsible for human phenotype, we can observe genes’ influences in every area of human functioning, also in sport. In this case, they are treated as a potential marker of predisposition and talent for specific sport disciplines. Genetic differences can result in varying functional and structural properties of muscles, such as their adaptation to effort, response to inflammatory processes, vulnerability to injuries and regeneration ability, and can affect even psychological traits that can be of high importance in different sport disciplines [9,10,11,12].

When considering personality traits, firstly, the evidence for the existence of an association between this polymorphic place and vulnerability to addiction was noticed, which is easy to understand as there exists a connection between the functioning of the dopaminergic system and addiction [13]. However, this association is more complex. Proper secretion of dopamine can also be considered as a factor influencing elementary personality traits, and therefore also the attitude towards sport and participation in it.

Our project is concentrated on the group of martial arts athletes; hence, we consider as reasonable analyzing polymorphisms in genes connected with dopamine secretion. To our best knowledge, there are many genes connected with dopamine secretion, potentially influencing personal attitude towards sport. Nonetheless, we concentrated on polymorphic areas in *DRD2 PROM.* rs1799732*, DRD2* rs1076560*, DRD2 Tag1D* rs1800498*, DRD2 Ex8* rs6276*, DRD2Tag1B* rs1079597 and *ANKK1 Tag1A* rs1800497. Dopamine, being a chemical molecule from the catecholamine group, is an important neurotransmitter secreted by dopaminergic neurons of the central nervous system. One of the traits influenced by this neurotransmitter is the attitude towards making “risky decisions” [14], as the main role of dopamine, and especially the isoforms of the D2 receptor, is integration of motivation, action and emotions. Hranilovic and coauthors observed that it seems to be of the highest importance in the case of novel stimuli. Knock-out of D2L influences the ability of exploration and the time that is needed to react in a harmful situation [15] Another study noticed the fact that there is a positive relation between mesocorticolimbic dopamine (DA) and the performance of learned reward-directed behavior [16,17].

*DRD2* and *ANKK1* gene polymorphisms influence individual differences in addiction and impulsiveness among people who do not practice sport. However, other studies indicate the existence of an association between the effectiveness of sequential, motor learning in the group of European women of Caucasian origin, an ability that can be of high importance for athletes as they have to accomplish complex sets of movements and present a high level of coordination [18].

## 2. Materials and Methods

### 2.1. Subjects

The research group included 258 male athletes (mean age = 26.02; SD = 8.30), whereas the control group was 284 healthy male volunteers matched for age (mean age = 22.89; SD = 4.78). All the participants were informed about purpose of the research and signed informed consent before the tests. Both the control and research group included individuals with Caucasian origin from the same region of Poland to reduce the possibility of racial gene skewing and to overcome any potential problems due to population stratification. The study was conducted among 258 Polish, healthy (no prior history of substance dependency or psychosis), male combat athletes aged 26.02 ± 8.30 (MMA, *n* = 83; judo, *n* = 67; boxing, *n* = 56; karate, *n* = 25; kickboxing, *n*= 24; wrestling, *n* = 3). Various methods were used to obtain the samples, including targeting national teams and providing information to national coaching personnel and athletes attending training camps.

The study was conducted according to the guidelines of the Declaration of Helsinki, and approved by KOMISJA BIOETYCZNA przy Okręgowej Izbie Lekarskiej w Szczecinie, ul. Marii Skłodowskiej-Curie 11, 71-332 (protocol nr 13/KB/VI/2016, 8 December 2016).

### 2.2. Genotyping

The genomic DNA was extracted from venous blood using standard procedures. Genotyping was conducted with the real-time PCR method.

A LightCycler^®^ 480 II System (Roche Diagnostic, Basel, Switzerland) was applied to perform the fluorescence resonance energy into the genotypic data. The data related to the DRD2 gene polymorphism were obtained under the following conditions: PCR was performed with 50 ng DNA of each sample in a final volume of 20 µL containing 2 µL reaction mix, 0.5 mM of each primer, 0.2 mM of each hybridization probe and 2 mM MgCl_2_, according to the manufacturer’s instructions, with initial denaturation (95 °C for 10 min) and then 35 cycles of denaturation (95 °C for 10 s), annealing (60 °C for 10 s) and extension (72 °C for 15 s). After amplification, a melting curve was generated by holding the reaction at 40 °C for 20 s and then heating slowly to a level of 95 °C. The fluorescence signal was plotted against temperature to provide melting curves for each sample.

### 2.3. Statistical Analysis

The *DRD2 PROM.* rs1799732*, DRD2* rs1076560*, DRD2 Tag1D* rs1800498*, DRD2 Ex8* rs6276*, DRD2Tag1B* rs1079597 and *ANKK1 Tag1A* rs1800497 genotypes’ distribution was tested according to Hardy–Weinberg equilibrium (HWE) with the HWE software https://wpcalc.com/en/equilibrium-hardy-weinberg/ (accessed on 3 June 2021).

The frequencies of genotypes and alleles of the *DRD2 PROM.* rs1799732*, DRD2* rs1076560*, DRD2 Tag1D* rs1800498*, DRD2 Ex8* rs6276*, DRD2Tag1B* rs1079597 and *ANKK1 Tag1A* rs1800497 polymorphisms in the analyzed groups were compared by the chi square test. All analyses were performed using STATISTICA 13 (Tibco Software Inc., Palo Alto, CA, USA) for Windows (Microsoft Corporation, Redmond, WA, USA).

## 3. Results

It was shown that the frequency distribution of *DRD2 PROM.* rs1799732 was not in accordance with the HWE. A statistical difference was found between the received frequency and the expected frequency in people practicing combat sports and in the control group. It was also shown that the frequency distribution of *DRD2Tag1B* rs1079597 was not consistent with the HWE in the control group.

The frequency distribution of *DRD2* rs1076560*, DRD2 Tag1D* rs1800498*, DRD2 Ex8* rs6276 and *ANKK1 Tag1A* rs1800497 was consistent with the HWE in the sports group and the control group. Additionally, the frequency of the *DRD2Tag1B* rs1079597 polymorphism in people practicing combat sports was consistent with the HWE (Table 1).

Differences in the frequency of the *DRD2Tag1B* rs1079597 gene polymorphism were found between people practicing combat sports and the control group (C/C 0.74 vs. 0.67; C/T 0.24 vs. 0.27; T/T 0.02 vs. 0.05; χ^2^ = 6.218; *p* = 0.045) and alleles (C 0.86 vs. 0.82; T 0.14 vs. 0.18; χ^2^ = 4.520; *p* = 0.034) (Table 2). The observed results indicate the fact that the carriers of the mentioned gene polymorphism are more likely to participate in sport disciplines connected with willingness to fight, and their personality is more predisposed to achieve the assumed goals.

The *DRD2 PROM.* rs1799732, *DRD2* rs1076560, *DRD2 Tag1D* rs1800498, *DRD2 Ex8* rs6276, *DRD2Tag1B* rs1079597 and *ANKK1 Tag1A* rs1800497 genotypes and allele frequencies in the studied sample did not differ between the analyzed groups (Table 2).

The *DRD2* rs1076560 genotypes and allele frequencies in the studied sample did not differ between the analyzed groups (Table 2).

## 4. Discussion

In our research, we observed the occurrence of a statistically higher frequency of *DRD2Tag1B* rs1079597 CC polymorphism and lower frequency of TT polymorphism in the group of martial arts athletes in comparison with the control group. Similar was the situation with allele C, which was statistically more frequent than allele T in the group of martial arts athletes in comparison with controls.

The individual behavior of a person is conditioned by variations in DNA sequence [19]. However, it also remains under the influence of environment, drugs, hormones and many other external influences. Commonly occurring SNPs are observed with a frequency of about 0.1% when we compare two different people. A similar situation is observed in relation to mapped VNTR, which can be found in a number of about 700 [20].

Since there exist polymorphisms in different position, exemplary in NHLH2 or MAO-A, the most important in relation to physical activity are the changes in other dopaminergic system components, including, among others, D2–D5 receptors, which are the main concern of this article [21,22]. There are various elements engaged in the process; however, we decided to concentrate on the most significant in relation to physical effort adaptations. Some studies noticed the relation between physical activity behaviors and endocannabinoid substances, whereas others emphasize the existence of the hypothalamic–pituitary–adrenal stress response axis or neuropeptides [23,24,25].

Such observations concentrate on neuronal processes that influence behavior and physical activity motivation, but there exists no one simple answer, as there exists some evidence suggesting a probable role of the mesolimbic reward pathway as an element of the motivation to be physically active [26,27]. More precisely, motivation to participate in physical activity is connected with the nucleus accumbens (NAc), the role of which is to translate motivation into aims that are directed behaviors [28]. Reward-related motivation has been linked with the level of both molecular and cellular changes in NAc responsiveness [29,30]. Dopamine signaling acting in the area of D1-like receptors is an initiating element of happenings within the cell, and particularly, the production of cyclic adenosine monophosphate (cAMP) [31]. However, this relation was observed in rodents, although the same situation probably happens in humans.

Some studies suggest the important role of the belief in free will, which influences conscious and unconscious information, affecting engagement in purposive behavior and feelings of agency. Nonetheless, people who are strongly concentrated on free will are more aware of intentional binding. This fact probably corresponds with stronger belief in free will, which is affected by dopaminergic pathways. However, some studies reject such observations, although others emphasize such probability [32,33,34,35].

The relation between sense of agency, as an element of personality, and dopamine is not still completely understood. Dopamine influences the response to attention-inducing stimuli, as well as reward-related stimuli, which highly change human behavior and personality [36]. As it is part of the motivational reward system [24,31], it can condition memory formation and motor functions. Moreover, dopamine is treated as an element influencing the process of error prediction during action, which can temporarily delay induced depression, but unpredicted time of reward can induce its activation [37]. Hence, the polymorphisms in dopamine genes not only influence the control of intentional acts but also influence sense of agency, which seem to be very important in personality creation. The activity of dopamine might change the temporal binding of action and event. As a result, an individual with a decreased dopamine level will not feel any profits when it comes to reward signaling [37].

## 5. Conclusions

Because we observed a high level of association between polymorphisms within genes connected with the dopaminergic system and attitude towards physical activity, we consider it important to look more deeply into this area of regulation. Personality is a key factor in changing attitudes towards physical activity, and is considered as a potential marker of a human’s attitude and behavior in this area, which can be very useful in understanding possible variation in physical activity and the willingness of an individual to achieve goals.

## Figures and Tables

**Table 1:** Hardy–Weinberg equilibrium of *DRD2 PROM*. rs1799732, *DRD2* rs1076560, *DRD2 Tag1D* rs1800498, *DRD2 Ex8* rs6276, *DRD2Tag1B* rs1079597 and *ANKK1 Tag1A* rs1800497 in combat sport and control groups.

		Observed (Expected)	Allele Frequency	χ^2^	*p* Value
*DRD2 PROM.* rs1799732					
Combat sport *n* = 258	GG	208 (202.4)	p allele freq (G) = 0.89 q allele freq (O) = 0.11	11.967	0.0005 *
	OO	9 (3.4)			
	GO	41 (52.3)			
Controls *n* = 284	GG	229 (225.4)	p allele freq (G) = 0.89 q allele freq (O) = 0.11	4.870	0.027 *
	OO	7 (3.4)			
	GO	48 (55.2)			
*DRD2* rs1076560					
Combat sport *n* = 258	CC	180 (176.7)	p allele freq (C) = 0.83 q allele freq (A) = 0.17	2.029	0.147
	AC	67 (73.6)			
	AA	11 (7.7)			
Controls *n* = 284	CC	199 (197.6)	p allele freq (C) = 0.83 q allele freq (A) = 0.17	0.346	0.556
	AC	75 (77.7)			
	AA	10 (7.6)			
*DRD2Tag1D* rs1800498					
Combat sport *n* = 257	AA	100 (95.3)	p allele freq (A) = 0.60 q allele freq (G) = 0.40	1.515	0.218
	AG	113 (122.4)			
	GG	44 (39.3)			
Controls *n* = 284	AA	101 (98.8)	p allele freq (A) = 0.59 q allele freq (G) = 0.41	0.294	0.588
	AG	133 (137.4)			
	GG	50 (47.8)			
*ANKK1 Tag1A* rs1800497					
Combat sport *n* = 258	GG	179 (181.7)	p allele freq (G) = 0.84 q allele freq (A) = 0.16	1.523	0.217
	AG	75 (69.6)			
	AA	4 (6.7)			
Controls *n* = 284	GG	195 (196.1)	p allele freq (G) = 0.83 q allele freq (A) = 0.17	0.221	0.638
	AG	82 (79.8)			
	AA	7 (8.1)			
*DRD2 Ex8* rs6276					
Combat sport *n* = 258	TT	116 (110.7)	p allele freq (T) = 0.66 q allele freq (C) = 0.34	2.967	0.144
	CT	106 (116.6)			
	CC	36 (30.7)			
Controls *n* = 284	TT	119 (117.3)	p allele freq (T) = 0.64 q allele freq (C) = 0.36	0.198	0.656
	CT	127 (130.4)			
	CC	38 (36.3)			
*DRD2Tag1B* rs1079597					
Combat sport *n* = 258	CC	192 (192.7)	p allele freq (C) = 0.86 q allele freq (T) = 0.14	0.1577	0.691
	CT	62 (60.5)			
	TT	4 (4.7)			
Controls *n* = 284	CC	195 (189.5)	p allele freq (C) = 0.82 q allele freq (T) = 0.18	4.7244	0.030 *
	CT	74 (85.0)			
	TT	15 (9.5)			

*p*—statistical significance, χ^2^—chi square test result, *n*—number of subjects, *—significant statistical differences.

**Table 2:** Frequency of genotypes and alleles of the *DRD2 PROM.* rs1799732, *DRD*2 rs1076560, *DRD2 Tag1D* rs1800498, *DRD2 Ex8* rs6276, *DRD2Tag1B* rs1079597 and *ANKK1 Tag1A* rs1800497 polymorphisms in combat sport and control groups.

Group	Genotypes			Alleles	
	*DRD2 PROM.* rs1799732				
	GG *n* (%)	OO *n* (%)	GO *n* (%)	G *n* (%)	O *n* (%)
Combat sport *n* = 258	208 (0.81)	9 (0.03)	41 (0.16)	457 (0.81)	59 (0.19)
Controls *n* = 284	229 (0.81)	7 (0.02)	48 (0.17)	506 (0.83)	62 (0.17)
χ^2^ *p* value	0.564 0.754			0.073 0.786	
	*DRD2* rs1076560				
	CC *n* (%)	AC *n* (%)	AA *n* (%)	C *n* (%)	A *n* (%)
Combat sport *n* = 258–516	180 (0.70)	67 (0.26)	11 (0.04)	427 (0.83)	89 (0.17)
Controls *n* = 284–568	199 (0.71)	75 (0.26)	10 (0.03)	473 (0.83)	95 (0.17)
χ^2^ *p* value	0.204 0.903			0.050 0.819	
	*Tag1D* rs1800498				
	AA *n* (%)	AG *n* (%)	GG *n* (%)	A *n* (%)	G *n* (%)
Combat sport *n* = 257–514	100 (0.39)	113 (0.44)	44 (0.17)	313 (0.61)	201 (0.39)
Controls *n* = 284	101 (0.36)	133 (0.47)	50 (0.18)	335 (0.59)	233 (0.41)
χ^2^ *p* value	0.668 0.716			0.410 0.521	
	*ANKK1 Tag1A* rs1800497				
	GG *n* (%)	AG *n* (%)	AA *n* (%)	G *n* (%)	A *n* (%)
Combat sport *n* = 258–516	179 (0.69)	75 (0.29)	4 (0.02)	433 (0.84)	83 (0.16)
Controls *n* = 284–568	195 (0.69)	82 (0.29)	7 (0.02)	472 (0.83)	96 (0.17)
χ^2^ *p* value	0.569 0.752			0.130 0.718	
	*DRD2 Ex8* rs6276				
	TT *n* (%)	CT *n* (%)	CC *n* (%)	T *n* (%)	C *n* (%)
Combat sport *n* = 258	116 (0.45)	106 (0.41)	36 (0.14)	338 (0.65)	178 (0.35)
Controls *n* = 284	119 (0.42)	127 (0.45)	38 (0.13)	365 (0.64)	203 (0.36)
χ^2^ *p* value	0.739 0.690			0.180 0.668	
	*DRD2Tag1B* rs1079597				
	CC *n* (%)	CT *n* (%)	TT *n* (%)	C *n* (%)	T *n* (%)
Combat sport *n* = 258	192 (0.74)	62 (0.24)	4 (0.02)	446 (0.86)	70 (0.14)
Controls *n* = 284	195 (0.67)	74 (0.27)	15 (0.05)	464 (0.82)	104 (0.18)
χ^2^ *p* value	6.218 0.045 *			4.520 0.034 *	

*p*—statistical significance, χ^2^—chi square test result, *n*—number of subjects, *—significant statistical differences.

## Data Availability

Not applicable.
